# Association between Childhood Diarrhoeal Incidence and Climatic Factors in Urban and Rural Settings in the Health District of Mbour, Senegal

**DOI:** 10.3390/ijerph14091049

**Published:** 2017-09-12

**Authors:** Sokhna Thiam, Aminata N. Diène, Ibrahima Sy, Mirko S. Winkler, Christian Schindler, Jacques A. Ndione, Ousmane Faye, Penelope Vounatsou, Jürg Utzinger, Guéladio Cissé

**Affiliations:** 1 Department of Epidemiology and Public Health, Swiss Tropical and Public Health Institute, P.O. Box, CH-4002 Basel, Switzerland; sokhna.thiam@swisstph.ch (S.T.); mirko.winkler@swisstph.ch (M.S.W.); christian.schindler@swisstph.ch (C.S.); penelope.vounatsou@swisstph.ch (P.V.); juerg.utzinger@swisstph.ch (J.U.); 2 University of Basel, P.O. Box, CH-4003 Basel, Switzerland; 3 Département de Géographie, Université Cheikh Anta Diop de Dakar, Dakar-Fann BP 25405, Senegal; aminaniang@orange.sn; 4 Centre de Suivi Ecologique, Dakar-Fann BP 15532, Senegal; ibrahima.sy@cse.sn (I.S.); jacques-andre.ndione@cse.sn (J.A.N.); 5 Département de Biologie Animale, Université Cheikh Anta Diop de Dakar, Dakar-Fann BP 25405, Senegal; fayeo@orange.sn

**Keywords:** diarrhoea, negative binomial regression, rainfall, seasonality, temperature, Senegal

## Abstract

We assessed the association between childhood diarrhoeal incidence and climatic factors in rural and urban settings in the health district of Mbour in western Senegal. We used monthly diarrhoeal case records among children under five years registered in 24 health facilities over a four-year period (2011–2014). Climatic data (i.e., daily temperature, night temperature and rainfall) for the same four-year period were obtained. We performed a negative binomial regression model to establish the relationship between monthly diarrhoeal incidence and climatic factors of the same and the previous month. There were two annual peaks in diarrhoeal incidence: one during the cold dry season and one during the rainy season. We observed a positive association between diarrhoeal incidence and high average temperature of 36 °C and above and high cumulative monthly rainfall at 57 mm and above. The association between diarrhoeal incidence and temperature was stronger in rural compared to urban settings, while higher rainfall was associated with higher diarrhoeal incidence in the urban settings. Concluding, this study identified significant health–climate interactions and calls for effective preventive measures in the health district of Mbour. Particular attention should be paid to urban settings where diarrhoea was most common in order to reduce the high incidence in the context of climatic variability, which is expected to increase in urban areas in the face of global warming.

## 1. Introduction

Diarrhoea is one of the major causes of child morbidity and mortality. For example, in 2015, it was estimated that more than half a million children under the age of five died from diarrhoeal diseases [1]. Nearly half of these deaths occurred in sub-Saharan Africa. Although child mortality due to diarrhoeal diseases has declined annually by 6.5% since the establishment of the UN Millennium Development Goal 4 (MDG4) in 2000, in 2015, 9% of all child deaths were still due to diarrhoeal diseases, and morbidity from diarrhoea remains unacceptably high [2]. Climate change impacts and will increasingly influence human health, and is expected to affect waterborne diseases, including diarrhoeal diseases [3]. Environmental change is a major risk factor for public and global health, including children’s health [4]. Children may experience greater risk of infectious diseases like diarrhoea due to rise in the average global surface temperature and rainfall. Diarrhoea is an important disease to study in this context because of its sensitivity to climatic parameters, and because children are particularly vulnerable to temperature variability as their immune system is not yet fully developed [5,6]. The primary impact of climate on society results from extreme weather events which are linked to changes in climatic variability to a greater extent than to changes in the mean values of climatic variables [7]. In the specific case of diarrhoea, climatic variability, especially rainfall and temperature variation, more generally impact diarrhoeal incidence through their effects on the growth of the various bacteria, protozoa, viruses and helminths that cause the infections of which diarrhoea is a symptom [6]. The effects of variation in rainfall and temperature on diarrhoea depend on the season in which the variation occurs [8,9].

The relationship between climate and diarrhoeal diseases is complex because of the myriad confounding variables and transmission routes that can affect the disease incidence and the fact that diarrhoea is caused by different pathogens [10,11,12,13]. Despite this complexity, evidence suggests that climatic factors, such as temperature and rainfall, are associated with the occurrence of diarrhoea; indeed an increase in temperature is associated with an increase in the incidence of diarrhoea [14,15,16,17,18,19]. However, previous studies exploring the effects of climatic factors on diarrhoea are inconclusive. One reason is that climatic data obtained from observing stations might contain measurement bias, as most of these monitoring sites are located in or in close proximity to urban areas, while temperature may show considerable variation even within cities [20]. Satellite remote sensing technologies offer new opportunities because of the broad spatial coverage [21]. To our knowledge only few studies used satellite remote sensing data to assess the effects of climatic factors on childhood diarrhoea. However, there is little evidence to support the hypothesis that the association between climatic factors and diarrhoeal incidence differs between rural and urban areas. Also, to date, no studies have assessed the relationship between climatic factors and diarrhoeal incidence using satellite remote sensing data in Senegal. This study aimed to fill these knowledge gaps. Moreover, the relationship between temperature variability and childhood diarrhoea remains to be explored, even though large temperature variability may stress children’s immune system and compromise their resistance to intestinal aetiological agents [22].

Results from a recent study conducted in Senegal showed that there is an increase in temperature during the cool season with significant inter-annual variation (–1.8 °C to 1.7 °C) and also during the warm season with a somewhat lower inter-annual variation (–1.7 °C to 1.0 °C) [23]. The study also showed that the variation in temperatures and heavy rainfall observed in Senegal matched scenarios put forward by the Intergovernmental Panel on Climate Change (IPCC). Hence, it is important to understand climate variability as a risk factor for infectious diseases, particularly diarrhoea. Against this background, Senegal remains a vulnerable country in view of the high presence of climate-sensitive diseases, including diarrhoea, but also because of rapid urbanization and an increasing frequency of extreme whether events, such as flooding in urban areas. This means that climatic variations particularly extreme events, must be considered when safeguarding people’s health and wellbeing.

The current study focusses on the coastal secondary city of Mbour and explores the association between childhood diarrhoeal incidence and climatic factors. In this part of Senegal, the cold dry season extends from December to March and is characterized by a high diarrhoea burden. This season offers conditions that favour the rapid spread of pathogens or viruses causing diarrhoea. The objectives of the study were, firstly, to determine the incidence of diarrhoea in children under the age of five years in urban and rural settings of Mbour; secondly, to examine the seasonal patterns of diarrhoea in both areas; thirdly, to statistically assess the relationship between diarrhoea incidence and daily land surface temperature (LST_Day_) and night land surface temperature (LST_Night_), average temperature (LST), temperature variability (defined as the difference between temperature LST_Day_ and LST_Night_), and rainfall; and, fourthly, to examine if the relationship with diarrhoea incidence and climatic factors differ between urban and rural settings using health surveillance data for childhood diarrhoea and satellite remote sensing data for the climate over a four-year period (January 2011 to December 2014).

## 2. Methods

### 2.1. Ethics Statement

The study protocol was approved by the *Comité National d’Ethique de la Recherche* (CER) of Senegal (reference no. 0l06/2015/CER/UCAD). As we used secondary health data on children under the age of five years from the government health facilities, written informed consent was obtained from the Director of the *Division du Système d’Information Sanitaire et Sociale* and the *Direction de la Planification, de la Recherche et des Statstiques* at the Ministry of Health of Senegal and from the chief medical officer of the health district of Mbour. All information gathered was handled confidentially. No biological specimens (stool, urine or blood samples) were collected.

### 2.2. Study Site and Associated Datasets

**Figure 1:**
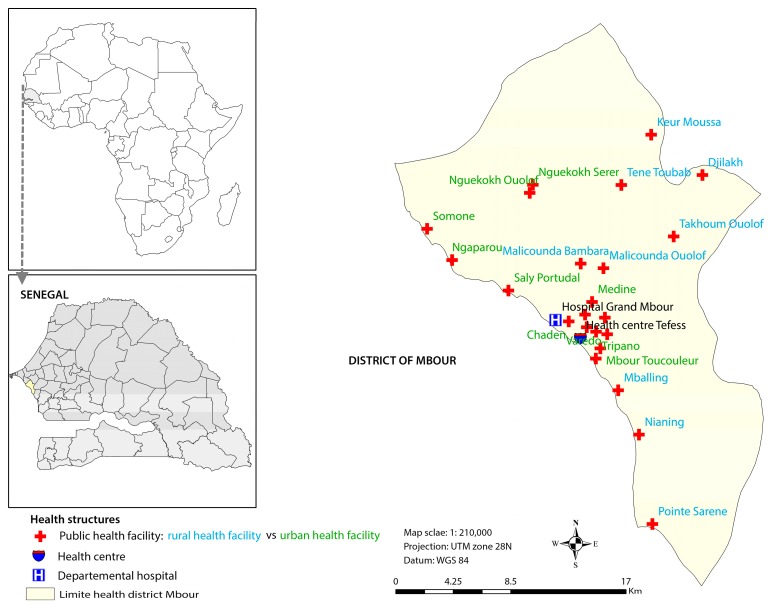
Map showing the study area and the location of the health facilities in the district of Mbour, Senegal.

Mbour is located in the western part of Senegal on the small coast, approximately 80 km south of the capital Dakar (Figure 1). The health district of Mbour consists of 23 posts and one health centre, which provide first-line health care services. Additionally, there is one hospital located in Mbour town.

Senegal has two main seasons: the rainy season (July–October with a peak in August–September) and the dry season (November–June). The dry season is divided in two seasons: the cold dry season (December–March) and the hot dry season (April–June and November). The mean annual temperature is between 22 °C and 30 °C, with monthly average in the hottest seasons of up to 35 °C, varying significantly between the coast and the interior of the country [24,25]. The relative humidity is high on the coast; it varies between 60% and 80%. The small coast of Senegal is vulnerable to weather events particularly flood-related health epidemics, drought, sea level rise and coastal erosion associated with climate change [25].

**Figure 2:**
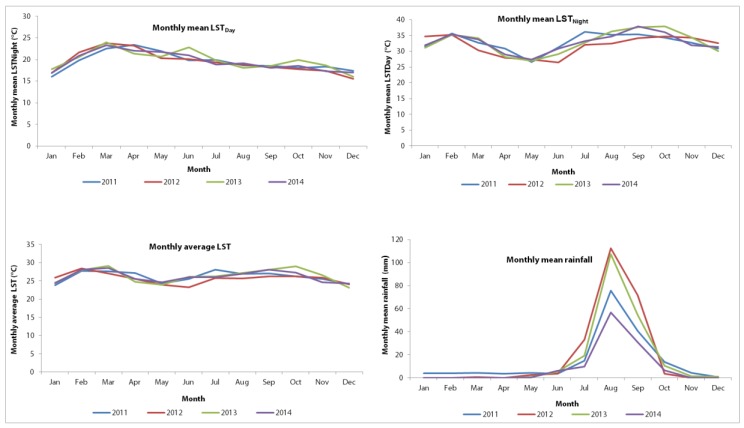
Distribution of mean monthly climatic variables in Mbour, Senegal over a four-year period from January 2011 to December 2014. Upper row: Distribution of mean monthly land surface temperature (LST_Day_) and Night (LST_Night_), by month and year, over the period 2011–2014. Lower row: Distribution of monthly average land surface temperature and monthly mean cumulative rainfall, by month and year, over the period 2011–2014.

In Mbour, the climate is tropical with an average annual precipitation of 496 mm. Figure 2 depicts key climate variables in the four-year study period. In brief, the monthly mean LST_Day_ and LST_Night_ were 32.6 °C and 19.5 °C, respectively. The mean monthly precipitation in the years 2011–2014 was 37.3 mm.

### 2.3. Data Sources

Table 1 summarizes the key health data (reported diarrhoea) and climatic variables, including reporting period and spatial resolution. Monthly records of diarrhoeal cases among children under the age of five presenting to government health facilities of the district of Mbour were obtained through the District Health Information System of the Ministry of Health of Senegal (DHIS_2_) from January 2011 to December 2014. For each child, information on age, sex, date of admission, name of the health facility visited and cause of the visit were provided. Diarrhoeal cases were aggregated by sex and age into two groups (<12 months and 12–59 months), as categorized by the DHIS_2_, and location of the health facility (urban and rural area). We were granted access to diarrhoea data through the District Data Manager of Mbour.

Monthly climatic data were obtained from readily available remote sensing data sources. In particular, we extracted day and night land surface temperature (LST_Day_ and LST_Night_) as a proxy of minimum/maximum air surface temperature from the Moderate Resolution Imaging Spectroradiometer (MODIS) satellite, and rainfall estimate (RFE) from the Africa Data Dissemination Service, covering the period 2011 to 2014. We validated these remote sensing data using an exploratory analysis provided as a supplementary file (Figure S1). We compared satellite data extracted from the health facility location that is closer to the single meteorological station present in our study area. The results showed that estimated LST_Day_, LST_Night_ and RFE were quite consistent and have similar pattern with the observed ground data from the meteorological station of Mbour.

### 2.4. Statistical Analysis

We analysed monthly health and climatic data from 24 health facilities (15 in urban and nine in rural settings) in the health district of Mbour for the period 2011–2014. A short-term time series analysis approach was first used to assess the seasonal pattern of diarrhoea and, secondly, to explore the association between diarrhoea and climatic variables. Several steps were involved in analysing the data in order to addresses our study objectives.

In a first step, a descriptive analysis was conducted to explore seasonal patterns of the climatic data and the number of diarrhoeal cases over time, as shown in Table 2. The proportions of diarrhoeal cases, means and standard deviations of climatic variables were compared across three seasons and by setting (urban vs. rural) according to a locally used seasonal calendar in Senegal, as shown in Table 1. Secondly, raw plots of mean monthly climate factors and monthly counts of diarrhoeal cases per health facility were generated and superimposed in order to visually check the seasonal pattern of climate-diarrhoea association over the four-year study period. Thirdly, to estimate effects of climatic parameters on monthly incidence of diarrhoea, we developed negative binomial regression models for monthly counts of diarrhoeal cases by health facility. These models included fixed intercepts for the different health facilities, for the calendar years and terms for seasonality, and they provided estimates of the incidence rate ratios (IRR) associated with the different predictor variables studied. To remove serial correlation of residuals, we added the lag 1 Pearson residual as further covariate to the model [26]. We conducted preliminary analyses to verify whether the climate–diarrhoea association was influenced by specifying climatic variables through different ways. We performed two separate models; one with continuous climatic variables and another one with categorical climatic variables to examine associations with diarrhoeal incidence.

Climatic variables such as LST, LST_Day_ and LST_Night_ were categorized into four groups defined by the quartiles of their values and indicating low, moderate, high and very high levels. Cumulative monthly rainfall was categorized into the following classes: ≤12 mm, 12–56 mm and ≥57 mm. Modification of the association between climatic variables and diarrhoea by location was evaluated, stratifying regression analyses between urban and rural areas. The quality of model fit was assessed based on the Akaike information criterion (AIC).

Negative binomial regression analyses were conducted separately in urban and rural areas and in both settings combined. All models were adjusted by using the number of under 5-year-old children outpatient of a given month as an offset. Moreover, they contained a calendar years trend, an indicator variable for type of setting (urban vs. rural) as well as terms describing seasonality. Seasonal patterns were described in two ways: (i) by considering three categories indicating cold dry season, hot dry season and rainy season, and (ii) by considering a cosine function: ƒ(*t*) = *α sin (2πt/12) + β cos (2πt/12)* where *t* is time measured in months. In addition to the values of temperature and rainfall of the same month, we also included the respective value of the preceding month to account for potential delays in the effect of temperature and rainfall on diarrhoeal incidence [6,27,28,29,30]. The reason for lagged climatic variables is to assess the sensitivity of diarrhoeal incidence to the potential delayed impact of weather event, as we expected diarrhoeal incidence to respond quickly to changes in rainfall and temperature due to the ubiquitous presence of bacteria [6]. Delayed effects are likely to be of lesser important for diarrhoea than, for example, malaria. Results are reported as IRR with 95% confidence intervals (CI) indicating the estimated relative change in incidence per unit increment in the respective predictor variable. All analyses were performed using Stata version 13.0 (Stata Corporation, College Station, TX, USA).

## 3. Results

### 3.1. Descriptive Analysis of Diarrhoeal Incidence and Climatic Data

A total of 111,302 child-visits were recorded in the 24 health facilities in the health district of Mbour in the four-year study period. The predominant cause for children under the age of five years visiting a health facility was acute respiratory infections (35,385 visits, 32.0%). Diarrhoea was the second most important cause for visiting a health facility (23,543 visits, 21.1%). Diarrhoeal cases ranged from 0 to 1306 cases with a mean of 20 cases per month. More than half of the cases (53.4%) were male. More than two-thirds of the cases were in the age group range of 12–59 months (69.1%). The incidence of diarrhoeal cases was higher in urban compared to rural settings (24.4% vs. 19.9%). The plots of monthly number of diarrhoeal cases indicated that diarrhoea peaked at the beginning of the year and after the mid-part of the year. The monthly average LST was 20.1 °C with monthly mean LST_Day_ of 32.6 °C (standard deviation (SD) 5.6 °C) and monthly mean LST_Night_ of 19.5 °C (SD 2.5 °C). A quarter (25.0%) of monthly observations had an LST_Day_ of 36 °C and above. The mean monthly cumulative rainfall was 14.8 mm (SD 28.5 mm) and 75.0% of the observations recorded less than 12 mm, whereas 10.0% of observations recorded more than 57 mm (Table 2).

### 3.2. Seasonal Patterns of Diarrhoeal Incidence in Urban and Rural Mbour

**Figure 3:**
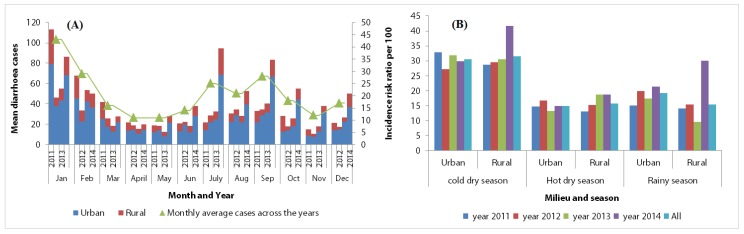
Distribution of mean monthly count of diarrhoeal cases per health facility in urban and rural settings in the health district of Mbour, Senegal over a four-year study period from 2011 to 2014. (**A**) Distribution of mean monthly diarrhoeal cases per health facility, by year, over the study period 2011–2014. (**B**) Distribution of diarrhoeal incidence (number of cases/population at risk times 100) across health facilities in urban and rural settings, and combined in Mbour over the three seasons.

Figure 3 shows the seasonal patterns of the mean numbers of diarrhoeal cases per month and facility from January 2011 to December 2014 in urban and rural settings of Mbour. A consistent pattern of seasonality in diarrhoeal incidence among children under the age of five years was observed in both areas.

There were two relative peaks in the number of diarrhoeal cases, as shown in Table 3 and Figure 3; one in the cold dry season with 42.7% of the reported diarrhoeal cases (10,048 cases), corresponding to the lowest mean temperature and lowest amount of rainfall; and another one in the rainy season, characterized by relatively warm temperature with 37.8% of the reported diarrhoeal cases (*n* = 8913). Throughout the study period, the burden of diarrhoeal disease was highest during the cold dry season with an average proportion of 30.5%. There was a corresponding trend in the rainy season with an average proportion of 17.9%. In the cold dry season, diarrhoeal cases were most likely to occur in January (17.6% of the cases) and February (11.8% of the cases) for the entire four-year period.

**Figure 4:**
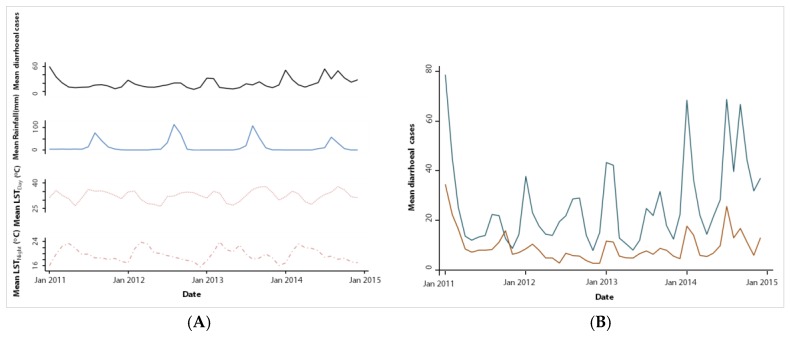
Trend in mean monthly mean diarrhoeal cases per health facility and climatic factors in the health district of Mbour over a four-year period from 2011 to 2014. (**A**) Trend in monthly mean diarrhoeal cases per health facility with mean rainfall, mean LST_Day_ and LST_Night_; (**B**) Trend in mean monthly diarrhoeal cases per health facility in urban and rural settings.

Table 4 and Figure 4 show that the numbers of diarrhoeal cases in rural settings were approximately five times lower than in urban settings (3895 vs. 19,648 cases). In 2014, an increase in the number of diarrhoeal cases was observed in the rural settings, both in the cold and rainy seasons, while this increase was only seen in the rainy season in the urban settings (Figure 4B). The overall incidence rate in both areas combined showed a significant difference by season (*p* < 0.001), age (*p* < 0.024) and sex (*p* < 0.001). Moreover, we observed a positive time trend in diarrhoeal cases.

Diarrhoeal incidence was found to be considerably higher in the cold dry season and in the rainy season (IRR: 2.03; 95% CI: 1.79–2.31; IRR: 1.84; 95% CI: 1.62–2.09, respectively), as illustrated in Table 5. In these two seasons, the risk of diarrhoea among children under the age of five years was higher compared to the hot dry season.

### 3.3. Association between Diarrhoea and Climatic Factors

The results of the unadjusted regression models are provided as a supplementary file (Table S1). The unadjusted analysis revealed significant positive associations between diarrhoeal cases and LST_Day_, average LST, LST variability and moderate rainfall in the same month (lag 0). It was found that a very high LST_Day_ ≥ 36 °C, average LST ≥ 28 °C and high LST variability ≥ 12 °C were significantly associated with higher number of monthly diarrhoeal cases (+25–95%), while moderate (13–56 mm) rainfall was associated with a somewhat higher number of diarrhoea (+18%). The unadjusted analysis revealed that moderate LST_Night_ at lag 0 showed a significant positive association with diarrhoeal incidence.

The results from the lagged models showed a significant negative associations between diarrhoeal incidence and high average LST ≥ 26 °C (IRR: 0.70, 95% CI: 0.58–0.86) and LST_Night_ ≥ 18 °C at one-month lag. High LST variability and high rainfall were also significantly positively associated with diarrhoeal incidence at one-month lag (IRR: 1.42, 95% CI: 1.16–1.75; IRR: 1.21, 95% CI: 1.01–1.45, respectively). In the multivariable analysis, association of diarrhoeal incidence with higher levels of LST_Day_, LST_Night_ and average LST of the same month (Table 6 and Table S2, respectively) were positive, while we did not find any evidence of an association. High LST_Day_ ≥ 32 °C and high LST ≥ 26 °C showed a significant negative association with diarrhoeal incidence of the following month (IRR: 0.78, 95% CI: 0.66–0.91; IRR: 0.76, 95% CI: 0.66–0.87, respectively).The corresponding results for LST_Night_ were even stronger. Compared to the lowest level, all other levels of LST_Night_ were associated with significantly lower diarrhoeal incidence in the following month. Moderate and high rainfall showed a significant positive association with diarrhoeal incidence in the same month (IRR: 1.23, 95% CI: 1.08–1.42; IRR: 1.34, 95% CI: 1.16–1.56, respectively).

We conducted a separate analysis to determine whether the relationship between diarrhoeal incidence and climatic factors differed in urban and rural settings (Tables S3 and S4). We found that in both settings, diarrhoeal incidence was positively associated with average LST ≥ 24 °C in the current month but not statistically significant when controlled for others variables; whereas in urban settings, a significant negative association was found between diarrhoeal incidence and average high levels of LST in the previous month (IRR: 0.79; 95% CI: 0.70–0.89; IRR: 0.73; 95% CI: 0.63–0.85) (Tables S3 and S4). We also found that high rainfall had a significant positive effect on diarrhoeal incidence in urban areas at lag 0 (IRR: 1.31; 95% CI: 1.12–1.54), while we found no such association in the rural setting. This suggests that the effect of climatic factors on diarrhoeal incidence differ between urban and rural settings in the health district of Mbour.

## 4. Discussion

To our knowledge, this is the first study using a time-series approach to quantify the association between diarrhoeal incidence in children under five years of age and climatic factors in an entire health district of Senegal. Health-related data were readily available from the DHIS_2_ for the four-year study period commencing in January 2011, while remotely sensed climatic data were obtained for the same time period from various data sources. Our study allowed examining the seasonal patterns of diarrhoeal incidence in the health district of Mbour.

We found two annual peaks in diarrhoeal incidence; a first peak occurred during the cold dry season (December–March), while a second peak was observed in the rainy season (July–October). The high number of diarrhoeal cases in the cold dry season is consistent with previous studies in Senegal [31,32]. Indeed, prior work in Senegal revealed that the cold dry season offers conditions that favour the rapid spread of pathogens or viruses causing diarrhoea [31,33]. Our findings are also in line with observations made in neighbouring Guinea Bissau [9] and Burkina Faso [34]. Viral infections causing diarrhoea were identified in previous studies during the cold dry season in children under the age of five years across large parts of sub-Saharan Africa, including Senegal [31,35,36,37,38,39,40]. These prior studies have reported prevalence rates due to rotavirus ranging from 18% to 41%. Importantly, rotaviral infections show the seasonal patterns in tropical climates [41,42]. Rotavirus is often the predominant aetiology of diarrhoea in infants and young children [43], with particularly high yields of isolation during the cooler months [44,45]. Although this may be an important explanation for the observed higher incidence of diarrhoeal illness during the cool dry season, the present study has not specifically investigated this association.

The second peak coincided with the rainy season and generally high temperatures, which mirrors the seasonal pattern of bacterial enteric infections [31]. We therefore speculate that during the rainy season, from July to October, the high number of diarrhoeal of infections might be driven by bacterial infection [31,34,38]. In Mbour, the rainy season includes the warmest months of the year, often coupled with floods, like in other parts of Senegal. The floods are partially explained by a poor drainage system. As a result, there is enhanced human contact with wastewater, which has been associated with cholera outbreaks [33]. Hence, it is conceivable that a large number of diarrhoeal cases during the rainy season results from an increased exposure to environmental pathogens and contaminated food due to high temperatures associated with accelerated bacterial growth [45]. However, there might be other reasons explaining the high rates of diarrhoea in these two seasons not investigated in the current study.

We found consistent diarrhoea seasonality in children under the age of five years in both urban and rural settings of Mbour, which calls for interventions and mitigation strategies of specific times of the year. The generally higher number of diarrhoeal cases found in the urban compared to rural settings, might be explained by overcrowding and lack of timely access to quality health care services.

The study revealed that, apart from seasonality, independent effects of temperature and rainfall were also associated with diarrhoeal incidence. Previous studies already reported positive associations between diarrhoeal incidence and temperature; indeed, an increase of temperature in the short-term (monthly or weekly) was associated with an increased risk of diarrhoea [6,46,47,48]. As reported in a prior study, pathogens causing diarrhoeal diseases respond differently to temperature variability [49]. The effect of high temperature on diarrhoeal incidence of the same month observed in the health district of Mbour may result from many factors. For instance, high temperature may lead to increased exposure to bacteria, parasites and other agents causing diarrhoea, implying that gastrointestinal diseases are more likely to occur during the hottest months of the year [14,50]. The associations of monthly temperatures (LST_Day_, LST_Night_, LST and LST variability) with diarrhoea incidence of the following month in the health district of Mbour were negative in the multivariate analyses. This suggests that the incubation period of the causative pathogen agent was shorter than one month.

Furthermore, our results from the multivariate analysis indicated that monthly mean cumulative rainfall has a positive effect on diarrhoeal incidence in the same month. Studies on the association between the risk of diarrhoea and rainfall have found contradictory results. In some cases, studies indicated that rainfall increases the risk of diarrhoea, which is in line with our observations [18,51,52]. Our findings are consistent with the results of Bandyopadhya et al., who found that rainfall and the prevalence of diarrhoea were positively associated across sub-Saharan Africa [6]. Other studies observed no association between rainfall and diarrhoea risk [16,19,53], even though a US study found that any rainfall four days prior was significantly associated with an 11% increase in acute gastrointestinal illness [54]. The positive association we found between diarrhoea and rainfall in the health district of Mbour could be explained by the fact that high rainfall can directly affect the transport of pathogens, and can affect the existing water and sanitation infrastructure, altering human exposure patterns [55]. The transport of pathogens resulting from heavy rainfall occurs in different ways. For example, if pathogens from animal or human excreta are present in soils and on environmental surface, rainfall can mobilize theses pathogens and transport them to surface water, exposing people to pathogens [56].

Our results showed that the effect of temperature (average LST) on diarrhoeal incidence was higher in rural compared to urban settings in the health district of Mbour but the difference was not statistically significant. In contrast, high rainfall was associated with a 31% increased risk of diarrhoea in urban settings, while there was no such association in rural settings. The reason why rainfall is a risk factor for diarrhoea in the urban setting may be due to higher levels of faecal contamination with higher exposure during the rainy season due to inexistent or unimproved sanitation system. Furthermore, in urban settings, the effects of overcrowding on diarrhoea may be exacerbated by high temperature associated with lower water availability for hygiene and sanitation. In the rural settings, high temperatures were associated with increased diarrhoeal risk, while rainfall showed no association. The negative association of LST_Day_ and average LST with diarrhoeal incidence of the following month observed in the urban settings is consistent with rotavirus seasonality studies [41].

This study provides evidence that the influence of temperature on diarrhoeal risk is more pronounced in rural than in urban settings, and rainfall is more likely to increase diarrhoeal risk in urban settings of Mbour. This may be explained by the interaction between climatic factors and differences in hygiene behaviour and sanitation status in urban and rural settings. The identification of climatic factors associated with diarrhoeal seasonality in this study sheds new light on the possible role of climatic variability in the occurrence of diarrhoea. Further, the potential factors of diarrhoeal seasonality we identified will facilitate future studies assessing the impact of social and economic development on diarrhoeal diseases in Senegal.

Several limitations should be acknowledged in this study. Firstly, our analysis relies on diagnosed diarrhoeal cases at health facilities, which, we assume, were mostly moderate or severe cases. Mild diarrhoeal episodes were more likely to go unreported. It follows that the trends reported here are only applicable to moderate and severe diarrhoeal cases. Secondly, our diagnostic work-up did not allow specific diagnosis of the pathogenic agents, which restrict us to examine the seasonality of specific pathogens, which might be necessary for future vaccine programmes. Given this shortcoming, we could not specifically analyse the association between climatic factors and the causative agents of diarrhoea. Characterizing the role of climatic factors in diarrhoeal risk is challenging due to a general lack of pathogen-specific diagnoses, with “all-causes” diarrhoeal data reflecting a combination of viral, parasitic and bacterial pathogens, which vary in transmission dynamics and sensitivities to environmental conditions [57]. With the mentioned limitations, the main strength of the current study is the use of an existing dataset (surveillance data) collected at 24 health facilities, coupled with remotely sensed climate data specific for health facility levels to assess the association between diarrhoeal diseases and climatic factors for a four-year period.

## 5. Conclusions

We found a seasonal fluctuation of diarrhoeal incidence in Mbour, western Senegal, characterized by a significant positive association of diarrhoeal incidence with rainfall and LST_Night_ in the same month among children under the age of five years, and a negative association between diarrhoeal risk and temperatures of the preceding month. We also found that diarrhoea is more associated with temperature in rural than in urban settings; while rainfall had no effect on diarrhoeal risk in the rural settings. Future studies should deepen our understanding of the association between diarrhoea and climatic factors, which is one of the main contributors to child mortality and morbidity in Senegal. Such knowledge will guide prevention and can lead programmes against diarrhoea. Our study indeed indicates that there is a need for effective preventive measures to reduce the high burden of diarrhoea in the health district of Mbour. Health intervention programmes in the cold dry season and in the rainy season focusing on morbidity control and prevention should be launched, particularly in urban settings where diarrhoea is most common, in order to reduce the incidence of diarrhoea in this context of climatic variability. We recommend that future studies should integrate detailed behavioural, climatic and socioeconomic factors, including relative humidity, access to water and sanitation, and more detailed surveillance data, including individual child data on diarrhoeal causation agents in order to improve the accuracy of predicting diarrhoeal morbidity in space and time. These factors should be included in the models to better explain the peak and trends in diarrhoeal occurrence as observed in Mbour.

## Figures and Tables

**Table 1:** Health data and remote sensing data sources, including reporting period and spatial resolution.

Data Source	Type	Period	Spatial Resolution
**Health Data**			
DHIS_2_	Number of visit to a health facility due to diarrhoea	1/2011–12/2014	
**Climatic Variables**			
MODIS	LST_Day_	1/2011–12/2014	1 × 1 km
MODIS	LST_Night_	1/2011–12/2014	1 × 1 km
USGS/Decadal RFE	RFE	1/2011–12/2014	80 × 80 km
SRTM-Altitude	-	-	90 × 90 m

DHIS_2_, District Health Information System of the Ministry of Health of Senegal; LST, land surface temperature; MODIS, Moderate Resolution Imaging Spectroradiometer (http://modis.gsfc.nasa.gov); SRTM-Altitude, Shuttle Radar Topography Mission (http://www.cgiar-csi.org/data/srtm-90m-digital-elevation-database-v4-1); USGS/RFE, United States Geographical Survey/Rainfall estimates (http://earlywarning.usgs.gov).

**Table 2:** Study parameters summarized for both areas combined (U/R), urban (U) and rural (R) in the health district of Mbour, Senegal and seasonal calendars extracted for the study period 2011–2014.

Climatic Variables * (Mean ± SD)		Cold Dry Season	Hot Dry Season	Rainy Season	Overall
		(December–March)	(April–June and November)	(July–October)	
**Monthly mean temperature**					
LST_Day_ (°C)	U/R	32.49 ± 3.89	29.78 ± 4.98	34.57 ± 5.71	32.64 ± 5.64
	Urban	32.45 ± 3.72	28.98 ± 4.26	33.89 ± 5.15	31.82 ± 4.87
	Rural	33.65 ± 5.18	31.11 ± 5.76	36.99 ± 7.07	33.97 ± 6.50
LST_Night_ (°C)	U/R	19.44 ± 2.79	20.62 ± 2.21	18.66 ± 0.98	19.59 ± 2.51
	Urban	19.84 ± 2.88	20.58 ± 1.89	18.64 ± 0.79	19.68 ± 2.19
	Rural	18.65 ± 3.38	20.70 ± 2.64	19.01 ± 2.28	19.45 ± 2.94
Average LST	U/R	26.15 ± 2.66	25.20 ± 2.52	26.93 ± 3.27	26.11 ± 2.92
	Urban	26.15 ± 2.43	24.77 ± 1.95	26.27 ± 2.60	25.74 ± 2.43
	Rural	26.16 ± 3.00	25.91 ± 3.13	28.00 ± 3.91	26.70 ± 3.49
Difference mean LST_Day_ and LST_Night_	U/R	13.54 ± 5.45	9.17 ± 5.86	16.29 ± 6.11	13.06 ± 6.50
	Urban	12.62 ± 4.56	8.42 ± 5.33	15.26 ± 5.21	12.15 ± 5.76
	Rural	15.00 ± 6.37	10.39 ± 6.48	17.98 ± 7.03	14.54 ± 7.30
Cumulative rainfall (mm)	U/R	1.00 ± 7.89	2.75 ± 6.62	37.30 ± 34.57	14.80 ± 28.51
	Urban	0.60 ± 4.35	1.78 ± 3.91	41.75 ± 36.81	14.71 ± 28.78
	Rural	1.52 ± 8.52	2.99 ± 8.11	40.42 ± 35.42	14.98 ± 28.05
**Diarrhoea proportion (%)**					
Proportion of diarrhoeal cases in outpatient children	U/R	30.67	15.11	18.49	21.10
	Urban	30.50	14.98	19.19	21.41
	Rural	31.46	15.72	15.40	19.95

* Monthly mean LST_Day_, LST_Night_, LST, difference LST and cumulative rainfall per health facility are presented; Urban = health facilities located in urban area; Rural = health facilities located in rural area; U/R = both areas combined; SD = standard deviation.

**Table 3:** Seasonal distribution of diarrhoeal cases for the period 2011–2014 in the health district of Mbour, Senegal.

Seasonal Calendar	Number of Diarrhoeal Case Visits	Percent (%)
Cold dry season (December–March)	10,048	42.7
Hot dry season (April–June and November)	4582	19.5
Rainy season (July–October)	8913	37.8
Overall	23,534	100.0

**Table 4:** Number of diarrhoeal cases and population at-risk among children under the age of five years per year extracted for the study period 2011–2014.

Year	Urban		Rural		Overall	
	Cases	Pop. At-Risk ^+^	Cases	Pop. At-Risk ^+^	Cases	Pop. At-Risk ^+^
2011	3947	14,053	1531	6511	5478	20,564
2012	3880	14,391	528	2181	4408	16,572
2013	4154	15,915	679	4186	4833	20,101
2014	7667	27,767	1157	2755	8824	30,522
Overall	19,648	72,126	3895	15,633	23,543	87,759

Urban = health facilities located in urban area; Rural = health facilities located in urban area; ^+^ The population at-risk as well as the number of cases was high in the last year of observation compared to the three earlier years based on the data received from the DHIS_2_.

**Table 5:** Effect of season on the number of visits of health care centred due to diarrhoea in the health district of Mbour, Senegal from 2011–2014.

Areas	Cold Dry Season		Rainy Season	
	IRR (95% CI)	*p-*Value	IRR (95% CI)	*p-*Value
Both areas combined	2.03 (1.79–2.31)	<0.001	1.84 (1.62–2.09)	<0.001
Urban	2.07 (1.80–2.37)	<0.001	1.91 (1.67–2.20)	<0.001
Rural	1.98 (1.50–2.632)	<0.001	1.70 (1.29–2.24)	<0.001

IRR: Incidence-rate ratio; 95% CI: Confidence interval, adjusted for clustering at the level of health facility.

**Table 6:** Results from the multivariate negative binomial regression models with climatic variables of the same and the preceding month, in the health district of Mbour, Senegal (January 2011–December 2014).

Adjusted Model			
Parameter		IRR (95% CI)	*p-*Value
**Residual lag 1**		1.04 (1.03–1.06)	<0.001
**Areas**			
	Rural	Ref.	
	Urban	1.53 (1.18–1.99)	<0.001
**Season**			
	Hot dry season	Ref.	
	Cold dry season	1.73 (1.55–1.92)	<0.001
	Rainy season	1.05 (0.90–1.22)	0.525
**Mean LST_Day_ (°C)**			
Lag 0	Low	Ref.	
	Moderate	1.02 (0.91–1.14)	0.734
	High	1.00 (0.87–1.16)	0.962
	Very high	1.00 (0.85–1.19)	0.968
Lag 1	Low	Ref.	
	Moderate	0.98 (0.88–1.10)	0.795
	High	0.87 (0.76–0.99)	0.043
	Very high	0.78 (0.66–0.91)	0.002
**Mean LST_Night_ (°C)**			
Lag 0	Low	Ref.	
	Moderate	1.13 (1.02–1.25)	0.014
	High	1.05 (0.94–1.17)	0.388
	Very high	1.11 (0.98–1.25)	0.089
Lag 1	Low	Ref.	
	Moderate	0.78 (0.71–0.87)	<0.001
	High	0.77 (0.69–0.87)	<0.001
	Very high	0.78 (0.68–0.91)	<0.001
**Mean cumulative rainfall (mm)**			
Lag 0	Low	Ref.	
	Moderate	1.23 (1.08–1.42)	0.003
	High	1.34 (1.16–1.56)	<0.001
lag 1	Low	Ref.	
	Moderate	1.03 (0.89–1.18)	0.716
	High	0.90 (0.77–1.05)	0.169
**Annual trend**			
	2011	Ref.	
	2012	1.21 (1.09–1.34)	<0.001
	2013	1.26 (1.14–1.39)	<0.001
	2014	1.38 (1.25–1.53)	<0.001

IRR: Incidence rate ratio; LST: Land surface temperature. Rainfall—low (≤12 mm), moderate (13–56 mm), high (≥57 mm); LST_Day_—low (<27 °C), moderate (27–32 °C), high (32–36 °C), very high (≥36 °C); LST_Night_—low (<18 °C), moderate (18–19 °C), high (19–21 °C), very high (≥21 °C); In this table, monthly mean LST_Day_, LST_Night_ and mean monthly cumulative rainfall in the same month (lag 0) and the previous month (lag 1) are presented. In addition to the variables presented, the model also included health facility and type of setting (i.e., urban vs. rural) as fixed factors and the lag 1 Pearson residual as further covariate.

## Supplementary Materials

The following are available online at www.mdpi.com/1660-4601/14/9/1049/s1, Figure S1: Comparison of satellite remote sensing data of monthly temperatures and rainfall extracted at the health facility location closest to the meteorological station with measured data from this station; Table S1: Results from the unadjusted negative binomial regression models with climatic variables of the same and the preceding month, in the health district of Mbour, Senegal (January 2011–December 2014); Table S2: Results from the adjusted negative binomial regression model with climatic variables of the same and preceding month, in the health district of Mbour, Senegal (January 2011–December 2014); Table S3: Results from the adjusted negative binomial regression model with climatic variables of the same and preceding month, in urban areas of district of Mbour, Senegal (January 2011–December 2014); Table S4: Results from the adjusted negative binomial regression model with climatic variables of the same and preceding month, in rural areas of the health district of Mbour, Senegal (January 2011–December 2014).
