# Supplementary material for: Association between Childhood Diarrhoeal Incidence and Climatic Factors in Urban and Rural Settings in the Health District of Mbour, Senegal
Source: Int J Environ Res Public Health. 2017 Sep 12;14(9):1049. doi: 10.3390/ijerph14091049 (PMC5615586; doi:10.3390/ijerph14091049)
Supplement: Supplementary file 1 [file ijerph-14-01049-s001.pdf]

Table S1. Results from the unadjusted negative binomial regression models with climatic variables of the same and the preceding month, in the health district of Mbour (January 2011- December 2014)

Parameter		Unadjusted model	
		Continuous	
		**IRR (95%CI)**	**P-value**
Average lst[a)]			
Lag 0	Low	Ref	
	Moderate	1.25 (1.04 – 1.72)	0.017
	High	1.02 (0.83 – 1.24)	0.860
	Very high	1.25 (1.00 – 1.55)	0.048
Lag 1	Low	Ref	
	Moderate	1.01 (0.84 – 1.22)	0.895
	High	0.70 (0.58 – 0.86)	< 0.001
	Very high	0.62 (0.49 – 0.77)	< 0.001
**Mean LST_{Day} (°C)**			
Lag 0	Low	Ref	
	Moderate	1.27 (1.06 – 1.52)	0.010
	High	1.54 (1.25 – 1.90)	< 0.001
	Very high	1.62 (1.29 – 2.03)	< 0.001
Lag 1	Low	Ref	
	Moderate	1.00 (0.83 – 1.21)	0.968
	High	1.11 (0.90 – 1.38)	0.323
	Very high	0.93 (0.74 – 1.18)	0.565
**Mean LST_{Night} (°C)**			
Lag 0	Low	Ref	
	Moderate	1.03 (0.88 – 1.21)	0.695
	High	0.73 (0.62 – 0.85)	< 0.001
	Very high	0.67 (0.57 – 0.79)	< 0.001
Lag 1	Low	Ref	
	Moderate	0.77 (0.66 – 0.90)	< 0.001
	High	0.76 (0.65 – 0.89)	< 0.001
	Very high	0.53 (0.45 – 0.62)	< 0.001
**LST variability[b)]**			
Lag 0	Low	Ref	
	Moderate	1.48 (1.25 – 1.75)	< 0.001
	High	1.95 (1.62 – 2.34)	< 0.001
	Very high	1.95 (1.59 – 2.40)	< 0.001
Lag 1	Low	Ref	
	Moderate	1.37 (1.16 – 1.62)	< 0.001
	High	1.57 (1.30 – 1.89)	< 0.001
	Very high	1.42 (1.16 – 1.75)	< 0.001
**Mean cumulative rainfall (mm)**			
Lag 0	Low	Ref	
	Moderate	1.18 (1.01 – 1.38)	0.035
	High	1.02 (0.85 – 1.23)	0.830
Lag 1	Low	Ref	
	Moderate	1.12 (0.96 – 1.31)	0.130
	High	1.21 (1.01 – 1.45)	0.037

IRR: Incidence-rate ratio; LST: Land surface temperature; a) Average of LST_{Day} and LST_{Night}; b) Difference LST_{Day} and LST_{Night}. Categories: LST_{Day} - low (<27), moderate (27-32), high (32-36), very high (>=36). LST_{Nigh}t - low (<18), moderate (18-19), high (19-21), very high (>=21).
LST-low (<24), moderate (24-26), high (26-28), very high (>=28). LST variability -low (<8), moderate (8-12), high (12-18), very high (>=18). Rainfall - low (<=12), moderate (13-56), high (>=57). The model also included health facility as fixed factors.

Table S2. Results from the adjusted negative binomial regression model with climatic variables of the same and preceding month, in the health district of Mbour (January 2011- December 2014).

Adjusted model			
**Parameter**		**IRR (95%CI)**	***P-value***
**Residual lag 1**		1.04 (1.03 – 1.06)	< 0.001
**Areas**			
	Rural	Ref	
	Urban	1.53 (1.17 - 1.99)	0.002
**Season**			
	Hot dry season	Ref	
	Cold dry season	1.76 (1.61 - 1.92)	< 0.001
	Rainy season	1.04 (0.90 - 1.21)	0.551
**Mean LST (°C)**			
Lag 0	Low	Ref	
	Moderate	1.08 (0.96 - 1.21)	0.198
	High	1.08 (0.96 - 1.22)	0.206
	Very high	1.05 (0.91 - 1.21)	0.529
Lag 1	Low	Ref	
	Moderate	1.01 (0.91 - 1.13)	0.870
	High	0.82 (0.73 - 0.92)	< 0.001
	Very high	0.76 (0.66 - 0.87)	< 0.001
**Mean cumulative rainfall (mm)**			
Lag 0	Low	Ref	
	Moderate	1.20 (1.05 - 1.38)	0.009
	High	1.25 (1.08 - 1.44)	0.003
lag 1	Low	Ref	
	Moderate	1.07 (0.93 - 1.22)	0.368
	High	0.92 (0.80 - 1.06)	0.273
**Annual trend**			
	2011	Ref	
	2012	1.21 (1.10 – 1.34)	< 0.001
	2013	1.24 (1.12 – 1.37)	< 0.001
	2014	1.39 (1.26 – 1.53)	< 0.001

IRR: Incidence-rate ratio; LST: Land surface temperature. Rainfall - low (<=12), moderate (13-56), high (>=57) LST-low (<24), moderate (24-26), high (26-28), very high (>=28).
In this table, results for average mean temperature and mean monthly cumulative rainfall in the same month (lag 0) and the previous month (lag 1) are presented. The model also included health facility and type of setting as fixed factors and the lag1 Pearson residual as further covariate

Table S3. Results from the adjusted negative binomial regression model with climatic variables of the same and preceding month, in urban areas of district of Mbour (January 2011- December 2014).

Multivariate analysis			
**Parameter**		**IRR (95%CI)**	***P-value***
**Season**			
	Hot dry season	Ref	
	Cold dry season	1.81 (1.64 - 2.00)	< 0.001
	Rainy season	1.04 (0.88 - 1.24)	0.615
**Mean LST (°C)**			
Lag 0	Low	Ref	
	Moderate	1.05 (0.93 - 1.19)	0.437
	High	1.08 (0.95 - 1.23)	0.237
	Very high	1.03 (0.87 - 1.21)	0.714
Lag 1	Low	Ref	
	Moderate	0.98 (0.87 - 1.11)	0.777
	High	0.79 (0.70 - 0.89)	< 0.001
	Very high	0.73 (0.63 - 0.85)	< 0.001
**Mean cumulative rainfall (mm)**			
Lag 0	Low	Ref	
	Moderate	1.12 (0.96 - 1.32)	0.154
	High	1.31 (1.12 - 1.54)	< 0.001
lag 1	Low	Ref	
	Moderate	1.03 (0.87 - 1.21)	0.725
	High	0.98 (0.83 - 1.15)	0.789
**Annual trend**		1.00 (1.00 – 1.01)	0.073
	2011		
	2012	1.17 (1.04 – 1.30)	0.006
	2013	1.15 (1.03 – 1.29)	0.011
	2014	1.24 (1.11 – 1.38)	< 0.001

IRR: Incidence-rate ratio; LST: Land surface temperature. Rainfall - low (<=12), moderate (13-56), high (>=57) LST-low (<24), moderate (24-26), high (26-28), very high (>=28).

In this table, results for average mean temperature and mean monthly cumulative rainfall in the same month (lag 0) and the previous month (lag 1) in urban areas are presented. The model also included health facility as fixed factor.

Table S4. Results from the adjusted negative binomial regression model with climatic variables of the same and preceding month, in rural areas of the health district of Mbour (January 2011- December 2014).

Multivariate analysis			
**Parameter**		**IRR (95%CI)**	***P-value***
**Season**			
	Hot dry season	Ref	
	Cold dry season	1.69 (1.41 - 2.02)	< 0.001
	Rainy season	1.12 (0.84 - 1.49)	0.439
**Mean LST (°C)**			
Lag 0	Low	Ref	
	Moderate	1.15 (0.91 - 1.46)	0.248
	High	1.08 (0.81 - 1.43)	0.610
	Very high	1.11 (0.82 - 1.51)	0.490
Lag 1	Low	Ref	
	Moderate	1.04 (0.81 - 1.34)	0.743
	High	0.93 (0.70 - 1.25)	0.660
	Very high	0.80 (0.58 - 1.10)	0.172
**Mean cumulative rainfall (mm)**			
Lag 0	Low	Ref	
	Moderate	1.30 (0.99 - 1.71)	0.056
	High	1.01 (0.74 - 1.37)	0.945
lag 1	Low	Ref	
	Moderate	1.08 (0.83 - 1.40)	0.549
	High	0.80 (0.60 - 1.07)	0.134
**Annual trend**			
	2011	Ref	
	2012	1.24 (0.98 – 1.55)	0.068
	2013	1.48 (1.19 – 1.85)	< 0.001
	2014	1.83 (1.48 – 2.26)	< 0.001

IRR: Incidence-rate ratio; LST: Land surface temperature. Rainfall - low (<=12), moderate (13-56), high (>=57) LST-low (<24), moderate (24-26), high (26-28), very high (>=28).
In this table, results for average mean temperature and mean monthly cumulative rainfall in the same month (lag 0) and the previous months (lag 1) in rural areas are presented. The model also included health facility as fixed factors.

Figure S1. Comparison of satellite remote sensing data of monthly temperatures and rainfall extracted at the health facility location closest to the meteorological station with measured data from this station

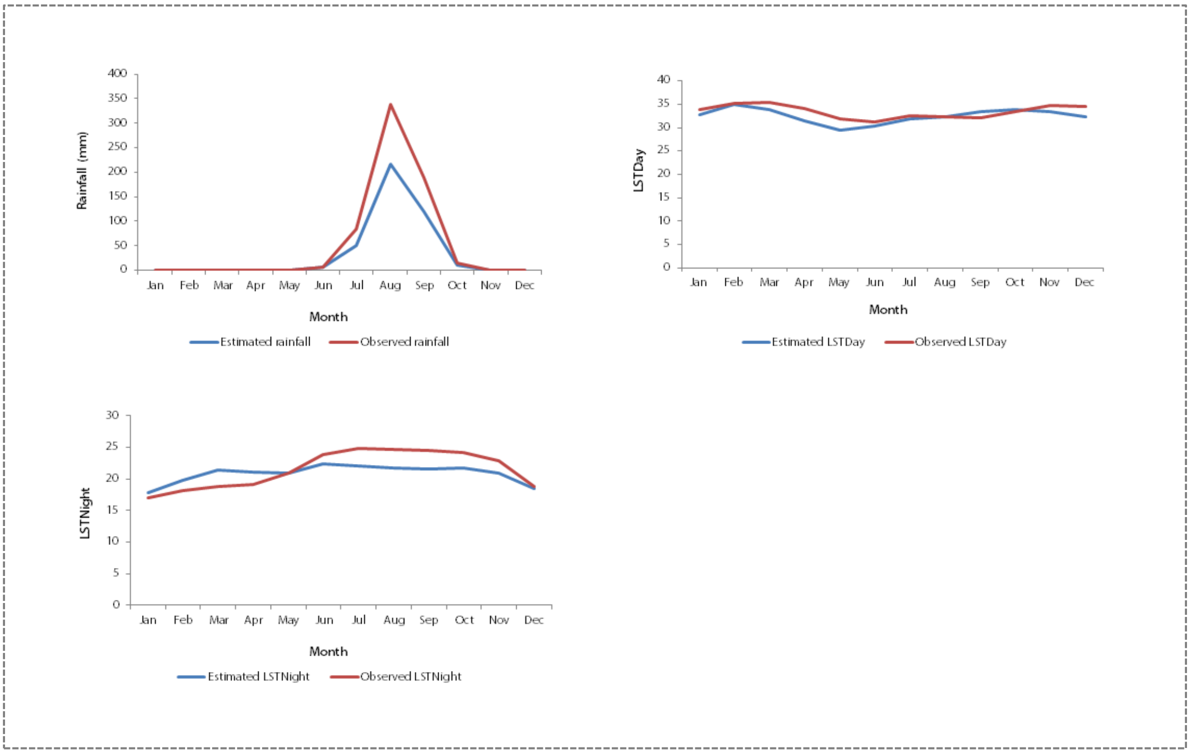

Rainfall (mm)
400
350
300
250
200
150
100
50
0
Jan Feb Mar Apr May Jun Jul Aug Sep Oct Nov Dec
Month
Estimated rainfall
Observed rainfall
LSTDay
40
35
30
25
20
15
10
5
0
Jan Feb Mar Apr May Jun Jul Aug Sep Oct Nov Dec
Month
Estimated LSTDay
Observed LSTDay
LSTNight
30
25
20
15
10
5
0
Jan Feb Mar Apr May Jun Jul Aug Sep Oct Nov Dec
Month
Estimated LSTNight
Observed LSTNight
